# Distribution dynamics and spatial dependence in healthcare resource allocation efficiency: evidence of path dependence and club formation in China

**DOI:** 10.3389/fpubh.2026.1811022

**Published:** 2026-03-31

**Authors:** Tong Lyu

**Affiliations:** School of Humanities and Arts, China University of Mining and Technology, Xuzhou, China

**Keywords:** dynamic evolution, healthcare system efficiency, provincial disparities, resource allocation structure, spatial Markov chain

## Abstract

**Background:**

The operational efficiency of the healthcare system is directly linked to the accessibility of health resources, the equitable distribution of health services across regions, and the sustainable functioning of the public health system. In the context of rapid population aging and growing medical demand, the persistent existence of interprovincial efficiency disparities may further intensify resource allocation imbalances and regional fragmentation in health service provision. Against this backdrop, this study examines the dynamic evolution and spatial interdependence of provincial healthcare system efficiency in China, systematically characterizing its transformation patterns and stratified structure.

**Methods:**

Using panel data from 31 Chinese provinces over the period 2001–2020, this study measures provincial healthcare system efficiency through an input-oriented Data Envelopment Analysis model under variable returns to scale. Pure technical efficiency is adopted as the core indicator, and efficiency states are categorized into low, medium, and high groups based on tercile thresholds. On this basis, Markov transition matrices and stationary distributions are constructed to capture efficiency mobility and long-term structural patterns. A spatial Markov framework is further employed to assess the influence of neighborhood efficiency contexts on state transitions. In addition, transition equations are estimated to identify structural factors associated with upward and downward efficiency movements.

**Results:**

During the sample period, the mean pure technical efficiency across provinces was 0.884, indicating the presence of residual input redundancy under the existing resource allocation structure. The Markov transition results reveal strong state persistence and limited cross-level mobility, reflecting pronounced path dependence. The stationary distribution further confirms the long-term coexistence of low-, medium-, and high-efficiency “clubs,” with corresponding steady-state probabilities of 44.91, 30.84, and 24.24%, respectively, suggesting no evidence of natural convergence. Spatial Markov analysis indicates that provinces located within high-efficiency neighborhoods are more likely to experience upward mobility, whereas clusters of low-efficiency regions tend to reinforce state persistence.

**Conclusion:**

China’s provincial healthcare system efficiency exhibits structural stratification and spatial interdependence, and efficiency disparities do not diminish automatically over time. From a policy perspective, greater emphasis should be placed on cross-regional coordination and the optimization of resource allocation structures. By facilitating upward efficiency mobility and mitigating the persistence of low-efficiency states, policymakers can reduce the long-term risk of structural divergence.

## 1 Introduction

In recent years, China has witnessed a sustained expansion in healthcare investment. According to official statistics, total health expenditure reached approximately RMB 8.53 trillion in 2022, accounting for about 7.05% of GDP, with overall spending remaining at a relatively high level (1). Meanwhile, the demographic structure has undergone profound transformation. By 2023, the population aged 60 and above had approached 300 million, representing more than 21% of the total population. Compared with the situation two decades ago, this marks a clear deepening of population aging and a corresponding rise in demand for healthcare services (2). Population aging continues to deepen, the prevalence of chronic diseases remains on the rise, and demand for healthcare services exhibits a long-term upward trend (3). Against this backdrop, governments at various levels have actively expanded medical resources, leading to substantial growth in the number of health professionals, hospital bed capacity, and the coverage of primary healthcare service networks (4). However, expansion in resource scale does not necessarily imply a corresponding improvement in allocation efficiency (5). Significant regional disparities in fiscal capacity, demographic composition, disease burden, and institutional environments have resulted in marked heterogeneity in the structural configuration, organizational arrangements, and utilization efficiency of healthcare resources (6). In the context of continuously rising health expenditure alongside persistent regional disparities, enhancing resource allocation efficiency has become a central issue in public health policy and health economics research. In such a context, improving allocation efficiency matters not only because health spending continues to rise, but also because greater investment does not automatically lead to better or more balanced service provision across regions (7).

From a health economics perspective, whether interregional differences in healthcare resource allocation efficiency diminish naturally over time carries important policy implications (8). If efficiency gaps merely reflect differences in development stages and gradually converge in the long run, regional disparities may primarily represent transitional adjustments (9). By contrast, if efficiency differences exhibit persistence or even structural entrenchment, this would imply systematic variation in the marginal returns to public fiscal expenditure across regions, potentially leading to unequal welfare outcomes and undermining the equity and sustainability of overall public health resource allocation (10). Within an institutional framework characterized by fiscal decentralization and uneven regional development, the formation of healthcare system efficiency depends not only on the scale of inputs but is also deeply embedded in resource structures, governance models, and institutional arrangements (11). It is therefore necessary to reassess the evolution of interprovincial efficiency disparities from a dynamic perspective, identifying whether they exhibit pronounced path dependence and whether they form stable stratified structures over time. This question is particularly relevant in China, where healthcare planning is nationally guided but largely implemented at the regional level. Against this institutional background, evidence on provincial differences in allocation efficiency can help identify persistent weaknesses and support better coordination across regions.

Existing studies on healthcare efficiency have largely employed Data Envelopment Analysis (DEA) or the Malmquist index to measure efficiency levels and changes across regions or periods, providing valuable evidence on regional disparities and technological progress (12). DEA has been widely applied in studies of health system efficiency, particularly in settings with multiple inputs and outputs, where the relationship between healthcare resources and service outcomes is difficult to capture with a single production function (13). However, such research typically focuses on cross-sectional comparisons of efficiency levels or changes in growth rates, with limited attention to the mobility of efficiency states across different intervals and their long-term distributional patterns (14). In other words, relatively few studies address a more structural question: are efficiency disparities characterized by high mobility and dynamic adjustment, or do they reflect stable hierarchical structures and persistent divergence? Moreover, China has long exhibited substantial spatial heterogeneity in regional development. The eastern, central, and western regions differ systematically in economic foundations, fiscal capacity, and healthcare delivery models (15). Similarities in institutional arrangements and resource allocation practices among neighboring regions may influence efficiency transitions through spatial spillovers and diffusion effects (16). If efficiency evolution demonstrates significant spatial conditional dependence, stratification may persist not only over time but also across space through clustering and reinforcement mechanisms (17). Accordingly, it is essential to integrate dynamic and spatial analytical frameworks to systematically examine state transitions and long-term stratification in provincial healthcare resource allocation efficiency in China, thereby deepening our understanding of the mechanisms underlying regional efficiency evolution and providing more structurally grounded policy implications for optimizing public health resource allocation.

## 2 Institutional background and analytical framework

### 2.1 Institutional features and resource allocation logic of China’s healthcare system

China’s healthcare system operates within an institutional framework characterized by government leadership alongside multi-level governance (18). Provincial governments play a pivotal role in fiscal arrangements, resource allocation, and institutional management (19). Substantial regional differences exist in fiscal capacity, demographic composition, disease profiles, and public service priorities. This institutional configuration gives rise to pronounced regional heterogeneity in the allocation of healthcare resources.

In terms of resource allocation logic, China’s healthcare system has long exhibited a pattern of “aggregate expansion alongside structural differentiation.” On the one hand, total health expenditure has continued to grow, accompanied by sustained expansion in the number of medical institutions, healthcare personnel, and hospital bed capacity (20). On the other hand, the distribution of resources across different tiers of healthcare institutions remains uneven (21). In some regions, resources are highly concentrated in large general hospitals, while primary healthcare capacity remains comparatively weak (22). In others, although primary care development has been prioritized, higher-tier medical resources are relatively insufficient (23). These structural differences imply that even when aggregate input levels are similar, regions may display significant variation in service delivery efficiency and resource utilization efficiency.

At the operational level, China’s healthcare system is shaped by both administrative governance logic and market-oriented incentive mechanisms (24). Medical service pricing, institutional entry, and staffing quotas remain subject to administrative regulation, while reforms in health insurance payment systems and rising medical demand have introduced elements of competition (25). Under such institutional conditions, resource allocation efficiency depends not only on technological factors but is also constrained by organizational models and institutional arrangements (26). Regional differences in the capacity to adjust resource structures and respond to institutional reforms may therefore contribute to the persistence of efficiency disparities over time.

### 2.2 Theoretical framework of efficiency dynamics, path dependence, and club formation

Against this institutional backdrop, the evolution of healthcare resource allocation efficiency is unlikely to follow a simple linear improvement trajectory; rather, it may exhibit inertia-driven dynamic adjustment (27). Existing resource structures, organizational arrangements, and institutional settings tend to display relative stability over extended periods, implying that current efficiency states can constrain the direction and magnitude of future changes (28). When the probability of state persistence is high, interregional efficiency rankings may remain largely unchanged over time, giving rise to path dependence (8).

From a dynamic perspective, efficiency evolution can be conceptualized as a process of state transitions across different efficiency intervals (29). Frequent cross-level upward transitions among low-efficiency regions would indicate a strong self-correcting capacity within the resource allocation system (27). Conversely, limited cross-level mobility suggests that efficiency gaps may gradually solidify, resulting in a stable hierarchical structure. When different efficiency levels coexist over a long period with limited mobility between them, this pattern can be described as the formation of “efficiency clubs” (9), a term used here to refer to a relatively stable stratification in efficiency dynamics rather than a convergence club in the strict econometric sense. Such a structure is not merely a static grouping but rather a stratified outcome shaped by high state persistence and constrained cross-level transitions throughout the dynamic process (30).

Moreover, in the presence of substantial regional spatial disparities, efficiency dynamics may be embedded within specific spatial contexts (29). Neighboring regions often share similarities in institutional arrangements, fiscal capacity, and governance practices, which may influence state transitions through mechanisms such as policy diffusion, experiential learning, and resource flows (16). If transition probabilities are systematically conditioned by the efficiency levels of surrounding regions, efficiency evolution would exhibit not only temporal path dependence but also spatial conditional dependence.

Building on this theoretical logic, this study develops an integrated analytical framework that combines efficiency measurement, state transition analysis, and spatial contextualization. First, provincial healthcare resource allocation efficiency is estimated using DEA. Second, Markov transition matrices are employed to characterize inter-state mobility and long-term distributional structures. Third, a spatial Markov approach is introduced to assess the extent to which neighborhood efficiency environments shape transition probabilities. This framework seeks to uncover, from both dynamic and spatial dimensions, the evolutionary trajectory and stratification mechanisms of provincial healthcare resource allocation efficiency in China.

## 3 Data and methodology

### 3.1 Data sources and variable construction

The data used in this study are drawn from the China Health Statistical Yearbook (2002–2021 editions), compiled by the National Health Commission of China. The yearbook aggregates administrative reports submitted by healthcare institutions at all levels and follows unified statistical standards with nationwide coverage, ensuring strong authority and temporal continuity. Compared with many self-reported datasets, this administrative reporting framework is better suited to long-term interprovincial comparison, as it is less affected by recall problems and sampling bias. Based on these data, we constructed a balanced panel dataset covering 31 provincial-level administrative regions in mainland China over the period 2001–2020. The provincial level is used here because it is the most consistent administrative scale for which comparable annual healthcare statistics are available throughout the sample period, and it is also a key level of healthcare planning and policy implementation in China. The year 2001 marks the earliest point at which the core indicators employed in this study became continuously available under a unified statistical framework. To avoid potential structural distortions associated with the COVID-19 pandemic particularly those affecting healthcare resource allocation and service utilization patterns data after 2020 were excluded from the analysis to preserve comparability and stability within the study period (31).

All variables are derived from provincially aggregated administrative statistics rather than survey-based samples, thereby reducing potential measurement bias arising from sampling errors. To ensure longitudinal consistency, all indicators were systematically cross-checked across yearbook editions. Where changes in statistical definitions or reporting standards were identified, variables were harmonized and backward-adjusted according to official methodological notes issued by the National Health Commission, ensuring temporal comparability. In terms of variable construction, the input variables include the number of practicing (assistant) physicians, registered nurses, and hospital beds, capturing healthcare resource inputs. These indicators are chosen because they reflect two core aspects of healthcare resource investment, namely human resources and medical infrastructure, both of which are central to the functioning of provincial healthcare systems (32). The output variables consist of outpatient visits and inpatient admissions, reflecting the scale of healthcare service provision. Together, these output indicators capture the realized volume of healthcare services and reflect the basic input–output relationship of provincial healthcare system performance. All indicators are based on provincial-level aggregated data. Descriptive statistics of the main variables are reported in Table 1.

**Table 1:** Descriptive statistics.

Variable	Unit	Mean	SD	Min	Max	Observations
Health personnel	Persons	280050.28	202201.55	10,058	1,027,917	589
Licensed physicians	Persons	70130.46	48245.92	2,885	271,412	589
Registered nurses	Persons	81543.71	66553.6	1,635	374,457	589
Beds per 1,000 population	Beds/1,000 persons	4.22	1.58	1.48	7.95	589
Medical operating expenditure	10,000 CNY	5901975.09	6210132.5	37017.2	42,792,293	527
Outpatient visits	Visits	76481974.32	68244073.36	2,010,742	401,317,285	589
Inpatient discharges	Persons	3725673.89	3227914.98	42,675	15,706,797	589
Doctor–nurse ratio	Ratio	0.96	0.24	0.58	2.09	589
Beds per staff	Beds/person	0.63	0.09	0.37	1	558
Primary care supply index	Index	5.26	2.83	0.85	19.47	505

### 3.2 Measurement of healthcare system efficiency

#### 3.2.1 DEA model specification

To measure provincial healthcare resource allocation efficiency, this study employs DEA. DEA is a non-parametric frontier approach widely used for evaluating relative efficiency under multiple-input–multiple-output conditions (29). It is particularly well suited to complex systems such as healthcare, where inputs are multifaceted and outputs cannot be adequately represented by a single functional form (33). Drawing on existing studies of healthcare efficiency assessment, this study adopts an input-oriented DEA model. This specification aligns with the practical concerns of public policy analysis namely, identifying the potential for input reduction while maintaining a given level of service output.

Given the substantial heterogeneity across Chinese provinces in terms of population size, healthcare demand, and institutional environments, the analysis incorporates the assumption of variable returns to scale (VRS) and applies the Banker Charness Cooper (BCC) model to estimate efficiency (34). This approach controls for scale effects and focuses on pure technical efficiency, which is primarily determined by managerial performance and resource allocation practices (35). In this study, PTE is treated as a DEA-based measure of relative efficiency rather than a parametric estimate with conventional standard errors. Within the conventional DEA projection framework, slack is reflected implicitly in the movement of decision-making units toward the efficient frontier. The analysis therefore retains the input-oriented BCC specification, rather than extending it to an SBM model, so that the efficiency scores remain comparable in the subsequent Markov and spatial Markov analysis. Input indicators cover key dimensions such as healthcare personnel and medical infrastructure, while output indicators reflect the actual utilization of healthcare services (36). Based on these specifications, annual efficiency scores for each province are calculated for the period 2001–2020, providing the empirical foundation for subsequent analyses of dynamic evolution and distributional patterns.

#### 3.2.2 Decomposition of technical and scale efficiency

To further identify the sources of efficiency loss, this study decomposes overall efficiency into its constituent components. In addition to the BCC model, which assumes VRS, the CCR model under constant returns to scale (CRS) is also estimated (36). The resulting comprehensive technical efficiency reflects the combined effects of managerial performance and scale factors, while scale efficiency is calculated as the ratio of CCR efficiency to BCC efficiency (37).

Table 2 presents the DEA estimation results for provincial healthcare resource allocation efficiency over the period 2001–2020. Under the input-oriented BCC model, the mean pure technical efficiency across the sample period is 0.884, indicating that, on average, provinces could reduce inputs by approximately 11.6% without decreasing service output. The mean comprehensive technical efficiency obtained from the CCR model is 0.853, suggesting that scale factors continue to constrain overall efficiency in some regions. The mean scale efficiency is 0.964, a relatively high level, implying that the operational scale of most provinces is close to optimal. Taken together, these results suggest that the primary bottleneck in provincial healthcare resource allocation efficiency does not stem from inappropriate scale, but rather from deficiencies in pure technical efficiency, involving managerial capacity, organizational structure, and resource allocation practices. This finding underscores the importance of moving beyond static efficiency measurement to examine the dynamic evolution and distributional characteristics of efficiency states.

**Table 2:** DEA efficiency summary.

Efficiency measure	Mean	SD	Min	Max
Pure technical efficiency (PTE, BCC)	0.884	0.083	0.61	1
Overall technical efficiency (TE, CCR)	0.853	0.094	0.562	1
Scale efficiency (SE)	0.964	0.041	0.781	1

### 3.3 Classification of efficiency states

To capture the dynamic mobility of interprovincial healthcare resource allocation efficiency, it is necessary to transform continuous efficiency scores into discrete state variables. The purpose of state classification is not to provide an absolute evaluation of efficiency levels, but rather to identify the relative position of each province within the overall distribution and to trace changes in that position over time. In this study, pure technical efficiency estimated under the BCC model serves as the basis for classification. Given that scale efficiency remains relatively high across the sample, PTE after controlling for scale effects better reflects differences in resource allocation structures and managerial capacity. Using PTE for state classification therefore facilitates a focus on the structural sources of shifts in efficiency tiers.

In determining the number of states, a balance must be struck between distributional resolution and estimation stability. Excessive subdivision may result in a sparse transition matrix, whereas too few categories may obscure meaningful differences in efficiency levels (38). Accordingly, efficiency is classified into three states: low, medium, and high. This three-state grouping keeps the classification easy to interpret while still preserving meaningful variation in provincial efficiency performance. Specifically, based on the distribution of PTE across all province–year observations in the sample period, the 33rd and 67th percentiles are used as threshold values (29). This percentile-based classification helps maintain comparability across provinces and over time by using a unified relative benchmark, while robustness is further checked through an alternative quartile-based grouping. Observations below the lower threshold are categorized as low-efficiency, those between the two thresholds as medium-efficiency, and those above the upper threshold as high-efficiency. The use of unified percentile thresholds ensures comparability across years and regions, allowing shifts in efficiency positions to be assessed under a consistent benchmark. To examine the sensitivity of the findings to the classification scheme, a quartile-based alternative is employed in the robustness analysis. The results indicate that the patterns of efficiency stratification and path dependence remain consistent under different classification standards. Based on this state definition, Markov transition matrices and stationary distribution models are subsequently constructed to characterize mobility probabilities across efficiency tiers and their long-term structural configuration.

### 3.4 Markov transition and club analysis

#### 3.4.1 Markov transition matrix

Building on the classification of efficiency states, this study further applies a Markov transition model to examine the dynamic evolution of provincial efficiency across different states. The Markov approach characterizes the temporal transition patterns of system states from a probabilistic perspective, making it particularly well suited for analyzing issues such as path dependence, state persistence, and long-term stratification (39). It has been widely employed in studies of regional economic development and efficiency dynamics.

Specifically, let $S_{t}\in \{1,2,3\}$ denote the efficiency state of a provincial healthcare system in year t, corresponding to low, medium, and high-efficiency states, respectively. The Markov transition probability $P_{\mathrm{ij}}$ represents the conditional probability that a province in state i at time t transitions to state j at time t + 1. Based on these transition probabilities, a three-state Markov transition matrix P can be constructed as follows:


$$P=(\begin{array}{ccc} p_{11} & p_{12} & p_{13}\\ p_{21} & p_{22} & p_{23}\\ p_{31} & p_{32} & p_{33} \end{array}),\sum_{j=1}^{3}p_{\mathrm{ij}}=1$$

Here, the diagonal elements $p_{\mathrm{ij}}$ capture the persistence or “lock-in” of efficiency states, whereas the off-diagonal elements describe the likelihood of transitions across different efficiency states. When low- or high-efficiency states exhibit high self-retention probabilities, it typically indicates pronounced path dependence in the evolution of efficiency.

Based on the transition matrix, the stationary distribution can be derived. The stationary distribution describes the long-run probability structure of the system residing in different efficiency states, thereby indicating whether efficiency tends to concentrate toward a particular tier or persists as a pattern of long-term stratified coexistence. The existence of a stationary distribution provides a dynamic basis for identifying efficiency “clubs,” rather than relying solely on cross-sectional differences.

#### 3.4.2 Long-term distribution and club formation

Based on the Markov transition matrix, the stationary distribution of efficiency states can be further derived. The stationary distribution characterizes the long-run probability structure of the system residing in different efficiency intervals under a limiting equilibrium, and its value lies in revealing the long-term configuration of efficiency tiers in the course of dynamic evolution rather than predicting the realized distribution in any specific period (40). If efficiency disparities exhibit a natural convergence tendency, the stationary probabilities would gradually concentrate on a single state. By contrast, if multiple efficiency intervals retain stable shares in the long run, this indicates that efficiency gaps do not dissipate over time but instead display persistent structural features.

When different efficiency states maintain relatively stable proportions in the stationary distribution and cross-tier mobility remains limited, the resulting pattern can be interpreted as the formation of “efficiency clubs.” Here, the term “club” does not refer to a static classification based on exogenous grouping; rather, it represents a dynamically stratified outcome jointly shaped by strong state persistence and constrained mobility. The boundaries across tiers remain stable over time, reflecting the long-term constraints imposed by resource allocation structures and institutional environments on efficiency evolution. This perspective grounds the identification of efficiency stratification in dynamic probability structures rather than relying solely on cross-sectional differences in any single period.

#### 3.4.3 Spatial Markov and conditional dynamics

Given the pronounced spatial disparities in regional development across China, efficiency evolution may not be an independent process but rather embedded within its surrounding neighborhood context (41). To examine whether efficiency dynamics exhibit spatial conditional dependence, this study extends the conventional Markov framework by incorporating a spatial Markov approach (42). Specifically, spatially lagged efficiency states are constructed based on geographic adjacency relationships, and different neighborhood contexts are defined according to the average efficiency levels of neighboring regions. Conditional transition matrices are then estimated separately under each neighborhood scenario.

The spatial Markov model does not aim to identify short-term causal effects; instead, it evaluates whether the neighborhood environment systematically alters the transition probabilities of efficiency states (43). If transition structures differ significantly across neighborhood contexts, this indicates the presence of spatial dependence in efficiency dynamics. High-efficiency neighborhoods may increase the likelihood of upward mobility, whereas clusters of low-efficiency regions may reinforce state persistence. By comparing the conditional transition matrices with the baseline Markov results, this approach provides a more comprehensive understanding of the structural characteristics of efficiency evolution across both temporal and spatial dimensions.

## 4 Results

### 4.1 Static efficiency patterns across provinces

**Figure 1:**
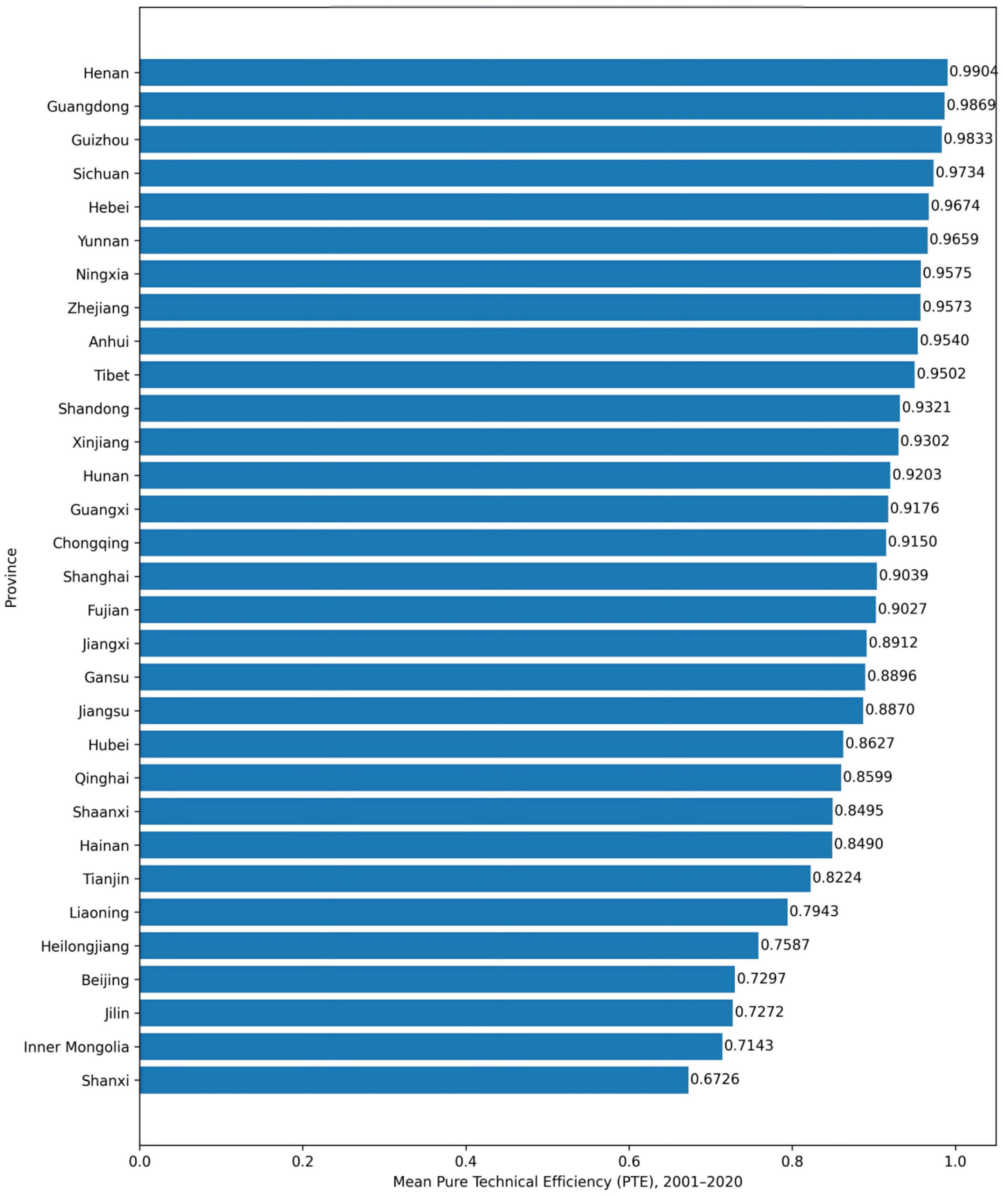
Provincial mean pure technical efficiency (2001–2020).

Figure 1 illustrates the long-term average levels of pure technical efficiency for each province from 2001 to 2020. Overall, the distribution reveals a relatively clear stratified pattern across provinces. A group of provinces has remained consistently close to the efficiency frontier, with mean PTE values approaching 1, reflecting relatively mature resource utilization capacity and managerial performance. Another group falls within a moderate efficiency range, with PTE generally clustered around 0.90. In contrast, a smaller number of provinces exhibit noticeably lower average efficiency levels, maintaining a considerable distance from the frontier.

Notably, interprovincial efficiency differences are not randomly dispersed but instead display a gradient pattern, forming relatively stable hierarchical relationships between high-performing and mid-to-low-performing regions. The gap between the provinces with the highest and lowest efficiency levels is substantial, indicating structural differences in technological utilization and operational performance. These disparities are not the result of short-term fluctuations; rather, they represent long-term averages accumulated over the sample period, suggesting a persistent pattern. Given that most provinces already operate at near-optimal scale, the observed efficiency differences are primarily driven by variations in pure technical efficiency rather than scale misallocation. In other words, efficiency improvement hinges less on adjusting input scale and more on enhancing resource utilization and managerial effectiveness.

In summary, the static analysis reveals a provincial efficiency landscape characterized by stable frontier regions, a clearly defined gradient structure, and enduring disparities. This structural differentiation provides an important foundation for subsequent analysis of efficiency evolution and regional stratification dynamics.

### 4.2 Dynamic evolution of efficiency states

Building on the classification of efficiency states, this study further examines the dynamic transitions of provincial efficiency across adjacent years. Table 3 reports the Markov transition probability matrix estimated from the provincial sample over the period 2001–2020.

**Table 3:** Markov transition matrix of healthcare system efficiency states.

From/To	Low efficiency (1)	Medium efficiency (2)	High efficiency (3)
Low efficiency (1)	0.8895	0.1105	0.0000
Medium efficiency (2)	0.1343	0.7214	0.1443
High efficiency (3)	0.0338	0.1498	0.8164

Elements are one-step transition probabilities from year t to t + 1 based on terciles of pure technical efficiency (PTE, BCC). Rows sum to one. The sample includes 31 provinces observed annually from 2001 to 2020, yielding 589 transitions.

Overall, all efficiency states exhibit relatively high persistence, with the diagonal elements of the transition matrix substantially larger than the off-diagonal entries, indicating strong temporal continuity in provincial healthcare resource allocation efficiency. Specifically, the self-retention probabilities for the low- and high-efficiency states reach 0.8895 and 0.8164, respectively, suggesting that provinces located at the two extremes of the efficiency distribution are more likely to remain in their original states in the subsequent year.

In terms of transition direction, movements occur primarily between adjacent tiers. The probability of transitioning from the low-efficiency state to the medium-efficiency state is 0.1105, while the probability of directly moving from low to high efficiency is zero, implying that large cross-tier leaps are highly unlikely. The medium-efficiency state exhibits both upward and downward mobility, with probabilities of 0.1443 for upward transitions to high efficiency and 0.1343 for downward transitions to low efficiency, highlighting its transitional role in the efficiency distribution.

Although the high-efficiency state demonstrates relative stability, it is not entirely immune to change. The probability of transitioning from high to medium efficiency is 0.1498, while the probability of directly falling to the low-efficiency state is comparatively small (0.0338). This suggests that high-performing provinces face some risk of efficiency fluctuation, even as they largely maintain their status.

Taken together, the Markov transition results indicate that provincial healthcare resource allocation efficiency in China exhibits pronounced path dependence and gradual evolution over the sample period. Efficiency dynamics are characterized more by persistence within existing tiers and incremental adjustments between adjacent levels than by frequent cross-tier mobility. These findings provide an essential basis for subsequent analysis of the long-term distributional structure and the emergence of efficiency “clubs.”

**Figure 2:**
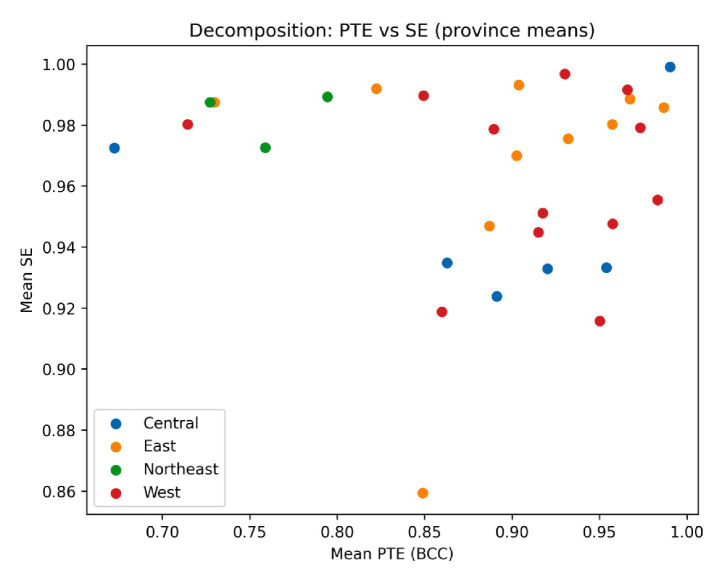
Decomposition of provincial efficiency: pure technical efficiency versus scale efficiency. Each point represents the provincial mean value over 2001–2020.

Examining overall efficiency levels alone is insufficient to uncover the sources of interprovincial disparities. It is therefore necessary to further explore the structural relationship between pure technical efficiency and scale efficiency. As illustrated in Figure 2, provinces display substantial variation across these two dimensions. Some regions exhibit relatively high pure technical efficiency but comparatively low scale efficiency, suggesting potential mismatches between input scale and service demand. Others demonstrate high scale efficiency but insufficient pure technical efficiency, indicating room for improvement in managerial capacity and resource utilization. In addition, several provinces perform poorly on both dimensions, implying more complex structural constraints in their efforts to enhance efficiency. These findings suggest that interprovincial efficiency disparities are not determined solely by the scale of resource inputs. Rather, they stem to a greater extent from differences in resource allocation structures and managerial performance. This structural heterogeneity provides an important empirical foundation for the subsequent Markov-based analysis of state transitions.

### 4.3 Club convergence and long-term stratification

Building on the Markov transition analysis, this study further examines the long-term distributional characteristics of provincial efficiency states in order to determine whether a stable stratified structure emerges in the process of efficiency evolution. Table 4 presents the stationary distribution derived from the Markov transition matrix.

**Table 4:** Stationary distribution of healthcare system efficiency states.

Efficiency state	Stationary probability (π)
Low efficiency (1)	0.4491
Medium efficiency (2)	0.3084
High efficiency (3)	0.2424

The stationary distribution π is obtained by solving π = πP, where.

The stationary results indicate that provincial efficiency does not converge toward a single state in the long run. Instead, a relatively stable distribution persists across different efficiency tiers. Under the stationary distribution, the low-efficiency state accounts for 44.91%, the medium-efficiency state for 30.84%, and the high-efficiency state for 24.24%. All three states maintain substantial proportions over time, suggesting that interprovincial efficiency stratification is a persistent phenomenon.

A closer examination reveals that the low-efficiency state occupies a dominant position in the long-term distribution, with a steady-state probability close to one-half of the sample, reflecting strong persistence. Although the share of the high-efficiency state is comparatively smaller, it remains at approximately 24%, indicating that a group of provinces is able to sustain relatively high performance over extended periods.

The medium-efficiency state, accounting for 30.84% in the stationary distribution, lies between the two extremes. In light of the transition analysis, this state primarily functions as an intermediate tier, with its long-term proportion shaped by the dynamic balance between upward mobility and downward transitions.

Overall, the stationary distribution demonstrates that China’s provincial healthcare system efficiency evolves into a configuration characterized by the coexistence of multiple efficiency clubs. Each club maintains relatively stable size and boundaries over the long term. Rather than converging toward a unified efficiency level, provincial performance exhibits the continued persistence of structural stratification.

P is the one-step Markov transition matrix. Probabilities sum to one.

**Figure 3:**
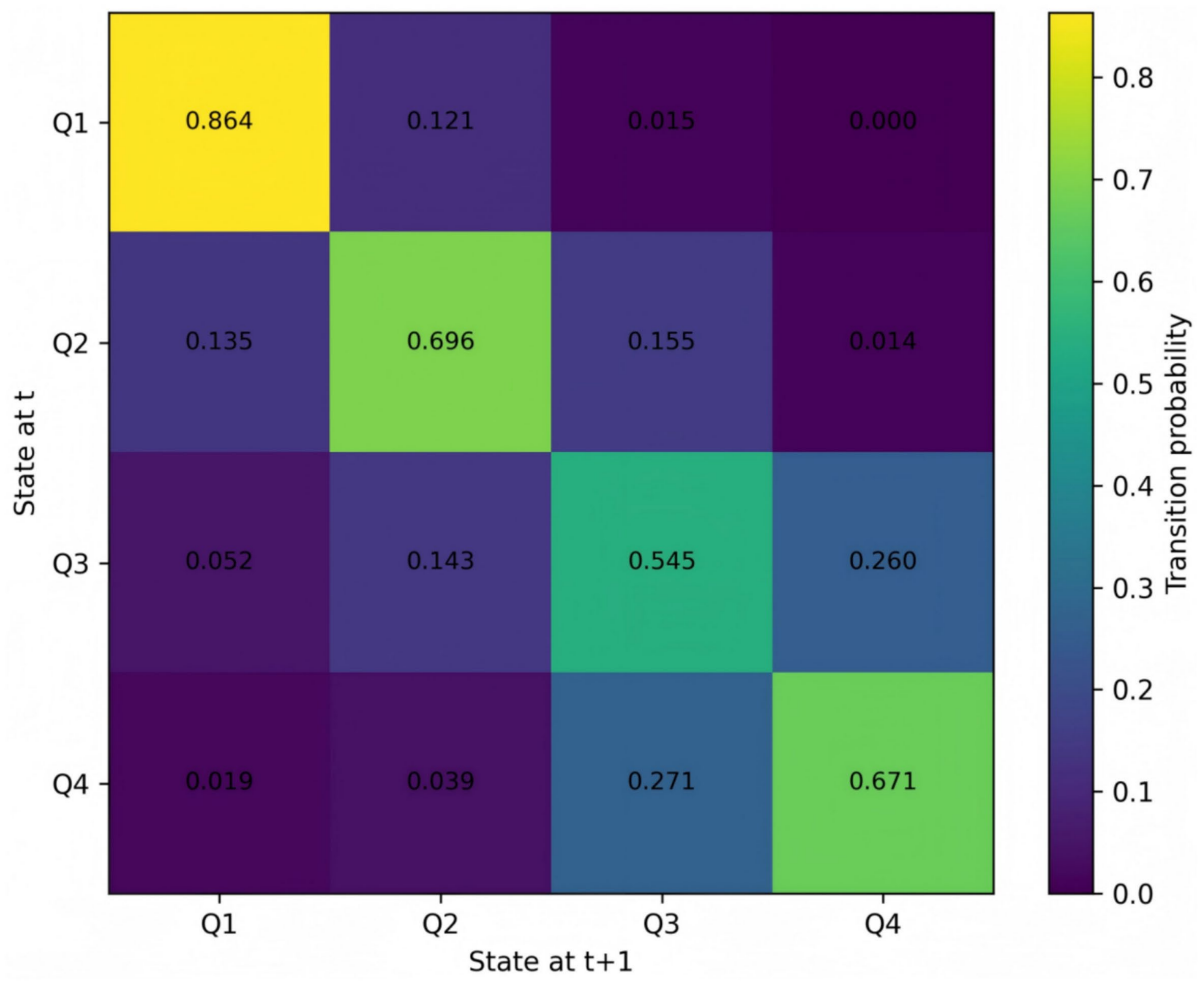
Quartile-based Markov transition matrix.

To examine whether the stratification results are sensitive to the state-classification scheme, this study extends the efficiency categorization from terciles to quartiles (Q1–Q4) and re-estimates the Markov transition matrix. The results are presented in Figure 3. Under this more refined classification, transition probabilities remain highly concentrated along the diagonal, and all quartile-based efficiency states continue to exhibit strong persistence, with cross-quartile mobility remaining limited. In particular, the lowest and highest quartiles display pronounced state retention, with no evidence of frequent cross-tier transitions. This robustness check shows that the observed patterns of efficiency stratification and path dependence are not driven by a particular threshold choice. Rather, they remain stable across alternative grouping schemes, further corroborating the long-term persistence of the efficiency club structure.

### 4.4 Regional and temporal heterogeneity in efficiency mobility

Building upon the overall Markov transition and stationary distribution analysis, this study further examines the heterogeneity of efficiency dynamics across regional and temporal dimensions. Tables 5, 6 report the Markov transition patterns and corresponding stationary distributions by region and by time period, respectively.

**Table 5:** Regional heterogeneity in efficiency dynamics (one-step Markov dynamics and stationary distribution).

Region	Transitions	p11	p22	p33	π_low	π_mid	π_high
East	190	0.9057	0.7143	0.7910	0.4472	0.3037	0.2491
Central	114	0.9333	0.8723	0.8649	0.4136	0.3599	0.2265
West	228	0.7660	0.6410	0.8155	0.3292	0.3523	0.3185
Northeast	57	0.9608	0.6667	–	0.8948	0.1052	0.0000

Diagonal elements (p11, p22, p33) represent state persistence. π_low, π_mid, and π_high denote stationary probabilities. Period-specific estimates are based on transitions where both t and t + 1 fall within the same subperiod.

**Table 6:** Period heterogeneity in efficiency dynamics (one-step Markov dynamics and stationary distribution).

Period	Transitions	p11	p22	p33	π_low	π_mid	π_high
2001–2005	124	0.8936	0.7353	0.9535	0.0924	0.1672	0.7404
2006–2010	124	0.8718	0.7778	0.6750	0.5543	0.3921	0.0536
2011–2015	124	0.8571	0.7436	0.9000	0.1053	0.2933	0.6015
2016–2020	124	0.9677	0.6170	0.6739	0.8992	0.0843	0.0165

Same definitions as Table 5. Spatially conditioned transition probabilities are reported by neighborhood category.

#### 4.4.1 Regional heterogeneity

From a regional perspective, substantial heterogeneity is observed in the dynamic evolution of efficiency (Tables 5). The eastern region demonstrates relatively strong state persistence, with self-retention probabilities of p11 = 0.9070, p22 = 0.7611, and p33 = 0.8375 for the low, medium, and high-efficiency states, respectively—each exceeding the national average. In the corresponding stationary distribution, the long-run share of the high-efficiency state in the eastern region reaches 30.87%, notably higher than the national figure of 24.24%.

In contrast, efficiency states in the central region exhibit weaker stability. The self-retention probability for the low-efficiency state is 0.8654, lower than that of the eastern region. The stationary distribution indicates that the low-efficiency state accounts for as much as 51.68% in the long run, while the high-efficiency state represents only 18.02%, suggesting persistent internal stratification within the region.

The western region displays transition characteristics that fall between those of the eastern and central regions. The self-retention probability for the low-efficiency state is 0.8821, and for the medium-efficiency state 0.7346. In the stationary distribution, the shares of low-, medium-, and high-efficiency states are 44.21, 32.15, and 23.64%, respectively, indicating a relatively balanced long-term structure.

The northeastern region, with a smaller sample size, exhibits highly concentrated state transitions. The self-retention probability for the low-efficiency state reaches 0.9412, and sustained high-efficiency states are rarely observed during the sample period. In the stationary distribution, the low-efficiency state dominates overwhelmingly, while the share of the medium-efficiency state remains limited.

#### 4.4.2 Stage heterogeneity

From a temporal perspective, the dynamic evolution of provincial efficiency exhibits pronounced stage-specific heterogeneity (Tables 6). During the period 2001–2005, the self-retention probability of the low-efficiency state was 0.8936. However, the stationary distribution indicates that the high-efficiency state accounted for 74.04%, while the low- and medium-efficiency states represented 9.24 and 16.72%, respectively. This suggests that in the early sample period, the efficiency distribution was more inclined toward a high-efficiency club configuration.

In 2006–2010, the self-retention probability of the low-efficiency state declined slightly to 0.8718. The stationary distribution shifted markedly, with the low-efficiency state rising to 55.43%, the medium-efficiency state accounting for 39.21%, and the high-efficiency state falling to 5.36%, indicating a concentration toward low- and medium-efficiency tiers.

During 2011–2015, the stationary shares of the low-, medium-, and high-efficiency states were 10.53, 29.33, and 60.15%, respectively, with the high-efficiency state once again becoming dominant. In contrast, in the period 2016–2020, the self-retention probability of the low-efficiency state further increased to 0.9677. The stationary distribution shows a substantial rise in the low-efficiency state to 89.92%, while the medium- and high-efficiency states declined to 8.43 and 1.65%, respectively. These results reflect a pronounced low-efficiency lock-in pattern in the later stage of the sample period.

### 4.5 Spatial Markov: conditional transition characteristics under neighborhood efficiency contexts

To further capture spatial dependence in the evolution of efficiency, this study constructs spatially lagged efficiency states based on geographic adjacency relationships and conducts a spatial Markov analysis accordingly. Specifically, the neighborhood efficiency indicator is defined as the average contemporaneous efficiency state of adjacent provinces. Based on tercile thresholds, neighborhood contexts are classified into low-, medium-, and high-efficiency environments. Conditional Markov transition matrices are then estimated separately under each context. Table 7 reports the spatial Markov transition results under different neighborhood efficiency scenarios.

**Table 7:** Spatial Markov transition matrices conditional on neighbor efficiency context.

Panel A. Low-neighbor context (Context 1)			
From/To	Low (1)	Medium (2)	High (3)
Low (1)	0.9059	0.0941	0.0000
Medium (2)	0.1591	0.6591	0.1818
High (3)	0.0435	0.1957	0.7609
Transitions in this context: 175			

Panel B. Medium-neighbor context (Context 2).			
From/To	Low (1)	Medium (2)	High (3)
Low (1)	0.8769	0.1231	0.0000
Medium (2)	0.1081	0.7838	0.1081
High (3)	0.0351	0.1404	0.8246
Transitions in this context: 196			

Panel C. High-neighbor context (Context 3).			
From/To	Low (1)	Medium (2)	High (3)
Low (1)	0.8710	0.1290	0.0000
Medium (2)	0.1446	0.6988	0.1566
High (3)	0.0288	0.1346	0.8365

Transitions in this context: 218. Entries are one-step transition probabilities from year t to t + 1, conditional on the neighbor efficiency context at time t. Rows sum to one. Neighbor context is defined using terciles of the spatial-lag state (neighbor_avg_state), with thresholds q33 = 1.8750 and q67 = 2.3333. Context 1/2/3 correspond to low/medium/high neighbor efficiency environments.

Focusing first on upward transitions from the low-efficiency state, systematic differences emerge across neighborhood contexts. Under the low-neighborhood scenario (Context 1), the probability of transitioning from low to medium efficiency is p12 = 0.0941. This probability increases to 0.1231 under the medium-neighborhood scenario (Context 2) and further to 0.1290 under the high-neighborhood scenario (Context 3). Correspondingly, the self-retention probabilities of the low-efficiency state are p11 = 0.9059, 0.8769, and 0.8710 across the three contexts, declining as neighborhood efficiency rises. These patterns indicate that in higher-efficiency environments, low-efficiency provinces are less likely to remain in place and more likely to experience upward mobility.

Turning to transitions from the medium-efficiency state, the probability of moving from medium to high efficiency also varies across contexts. Under the low-neighborhood scenario, p23 = 0.1818; under the medium-neighborhood scenario, it falls to 0.1081; and under the high-neighborhood scenario, it rises again to 0.1566. Meanwhile, the self-retention probabilities of the medium-efficiency state are p22 = 0.6970, 0.7568, and 0.7358, respectively, indicating differences in stability across neighborhood conditions.

Regarding the persistence of the high-efficiency state, provinces exhibit stronger stability when embedded in higher-efficiency neighborhoods. The self-retention probabilities for the high-efficiency state are p33 = 0.7609, 0.8246, and 0.8365 under low, medium, and high-neighborhood contexts, respectively, increasing with neighborhood efficiency levels. This suggests that high-efficiency states are more likely to be sustained within high-efficiency clusters, reflecting spatial clustering and self-reinforcing dynamics in efficiency evolution.

**Figure 4:**
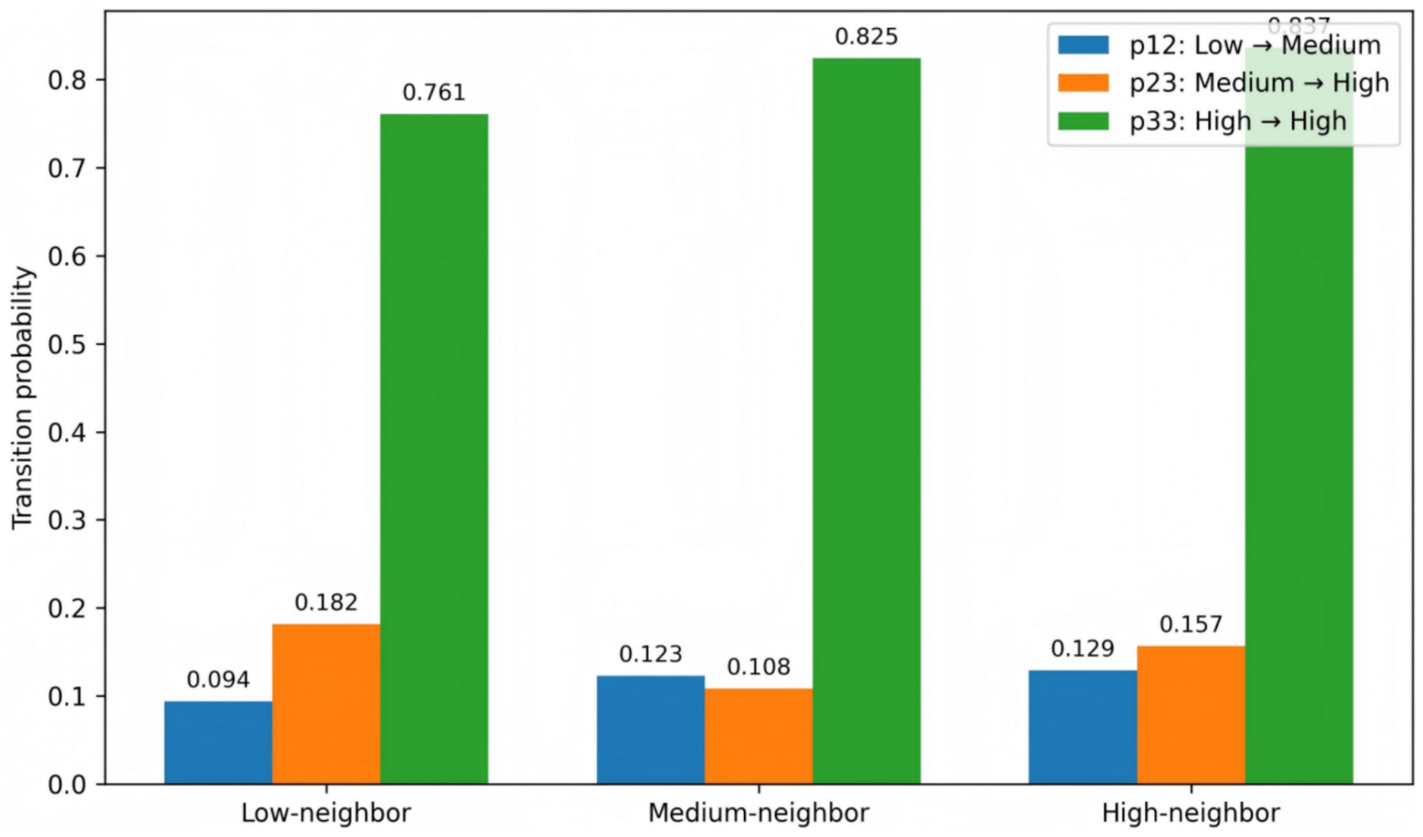
Key transition probabilities under different neighbor contexts.

Overall, the spatial Markov results indicate that the dynamic evolution of provincial efficiency exhibits pronounced spatial conditional dependence. On the one hand, higher neighborhood efficiency levels are generally associated with a greater probability of upward mobility for low-efficiency provinces. On the other hand, high-efficiency states are more stable within high-efficiency clusters. These findings provide additional empirical support, from the perspective of conditional spatial transitions, for the formation and persistence of efficiency clubs. Figure 4 visually presents the differences in key transition probabilities across neighborhood contexts, further illustrating the spatially embedded nature of efficiency dynamics.

It should be emphasized that the spatial dependence identified through the spatial Markov analysis is not intended to capture short-term causal spillover effects. Given the provincial scale of the analysis, the observed neighborhood differences are more likely to reflect institutional and governance spillovers—such as the diffusion of healthcare governance models, policy learning, and interregional institutional emulation—rather than cross-provincial patient mobility. This interpretation is consistent with the institutional context of China’s healthcare system, which is characterized by territorial administration and an emphasis on coordinated regional development.

**Figure 5:**
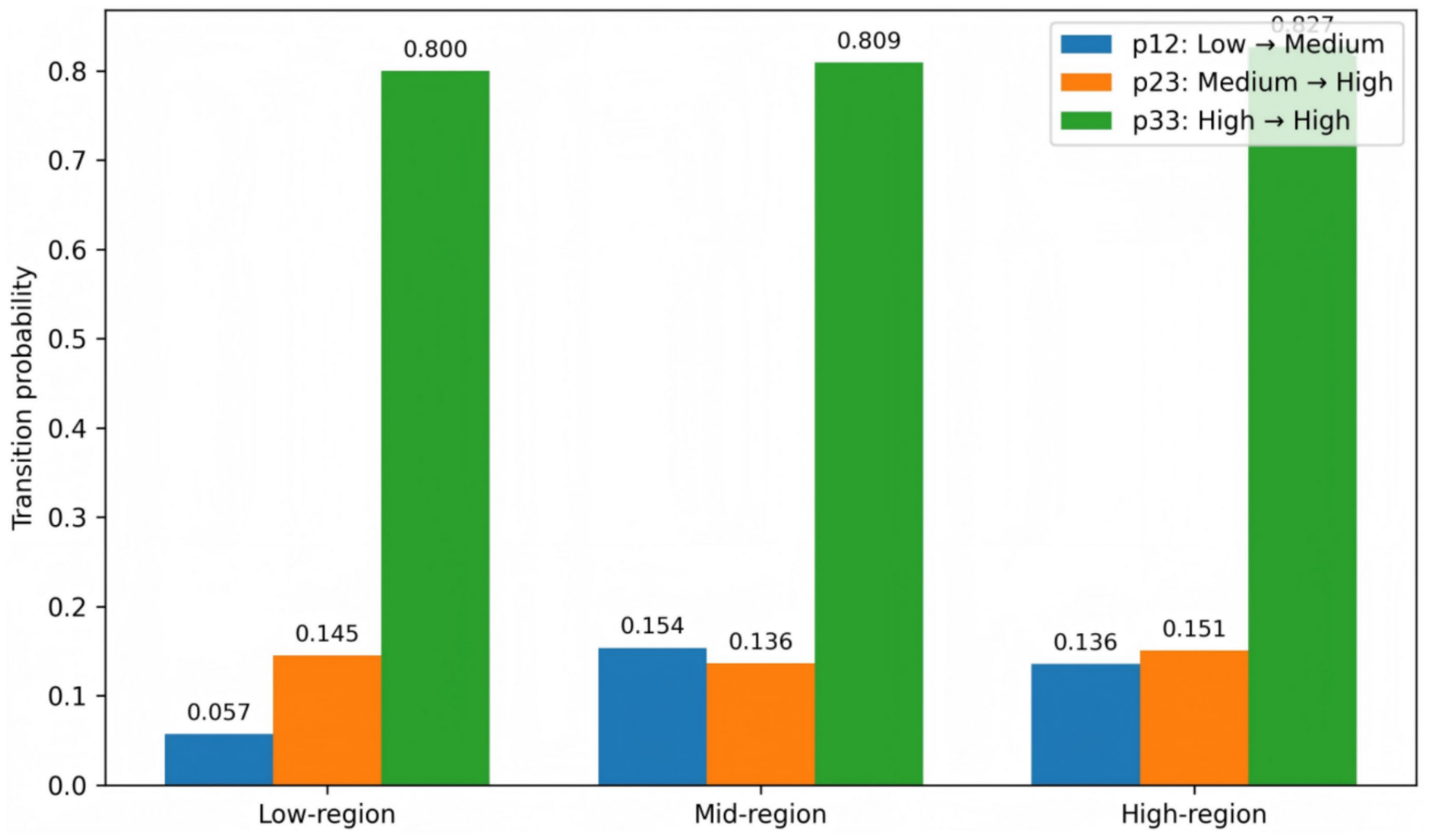
Key transition probabilities by same-region context.

As a robustness check, this study further reconstructs the spatial Markov model by defining neighborhood contexts based on within-region adjacency rather than geographic contiguity. The results show that the key transition probabilities across different neighborhood efficiency scenarios exhibit patterns consistent with the baseline findings (Figure 5).

### 4.6 Transition equations: determinants of efficiency state mobility

Building upon the spatial Markov findings that highlight significant spatial conditional dependence in efficiency transitions, this study further estimates transition equations to examine factors associated with upward and downward movements across efficiency states. It should be emphasized that these transition equations are not intended to identify causal mechanisms underlying efficiency changes. Rather, they serve as a complementary analysis to the Markov and spatial Markov results, capturing systematic associations between resource allocation structures, service burdens, and the likelihood of efficiency state transitions. Given that the transition variables are derived from prior DEA estimations, causal interpretations are deliberately avoided. Specifically, upward and downward transitions are treated as dependent variables in separate linear probability models. The estimation results are summarized in Table 8.

**Table 8:** Transition equations for efficiency state mobility.

Variable	Upward transition (low/medium → higher)	Downward transition (medium/high → lower)
Doctor–nurse ratio	0.1293 (0.1662)	0.0645 (0.1087)
Staff per 10 k	0.0011 (0.0014)	0.0036** (0.0018)
Beds per staff	1.1050*** (0.2713)	−0.0717 (0.1398)
Ln (GDP per capita)	−0.2124** (0.0894)	−0.0650 (0.0683)
Year fixed effects	Yes	Yes
Region fixed effects	Yes	Yes
Clustered standard errors	Province	Province
Observations	360	389
R-squared	0.2266	0.3493
Outcome definition	1 if state (t + 1) > state_t	1 if state (t + 1) < state_t

Coefficients are from linear probability models. Standard errors clustered at the province level are in parentheses. Significance levels: ***p < 0.01, **p < 0.05, *p < 0.10. Models include year fixed effects and macro-region fixed effects (East/Central/West/Northeast).

#### 4.6.1 Upward transition

In the upward transition model, indicators of medical resource structure and economic development exhibit significant associations with efficiency mobility. In particular, the ratio of hospital beds to health personnel (beds per staff) has a positive and statistically significant effect on the probability of upward transition, with a coefficient of 1.1050, significant at the 1% level. This suggests that, holding other factors constant, provinces with a higher intensity of bed allocation are more likely to experience upward efficiency shifts. At the same time, the logarithm of per capita GDP is significantly negatively associated with upward transition probability, with a coefficient of −0.2124 (significant at the 5% level). This indicates that provinces with higher levels of economic development are, ceteris paribus, less likely to exhibit upward efficiency mobility between adjacent periods. By contrast, neither the physician-to-nurse ratio nor the number of health personnel per 10,000 population reaches statistical significance in the upward transition model. After controlling for other factors, these two indicators do not display a significant linear association with upward efficiency transitions.

#### 4.6.2 Downward transition

The results of the downward transition model indicate that different resource allocation indicators play distinct roles in efficiency decline. The number of health personnel per 10,000 population has a positive and statistically significant effect on the probability of downward transition, with a coefficient of 0.0036 (significant at the 5% level). This suggests that provinces with higher personnel density are more likely to experience a decline in efficiency in the subsequent period. In contrast, neither the beds-per-staff ratio nor the physician-to-nurse ratio reaches statistical significance in the downward transition model, indicating that these structural indicators do not exhibit a stable linear association with efficiency decline. Similarly, the logarithm of per capita GDP does not show a significant effect in the downward transition specification.

## 5 Discussion

Within the DEA and Markov together with the spatial Markov framework, this study systematically investigates the structural evolution of provincial healthcare resource allocation efficiency in China from 2001 to 2020 across both dynamic and spatial dimensions. In contrast to existing studies that emphasize static comparisons of efficiency levels, the present analysis shifts the focus toward transition probabilities, state persistence, and stratified structures over time and space. By situating efficiency within a long-term evolutionary framework, the study provides new empirical evidence for understanding structural changes in the performance of China’s healthcare system.

The Markov transition results indicate pronounced path dependence in provincial healthcare efficiency. Provinces in low, medium, and high efficiency states all display high self-retention probabilities between adjacent years, while cross-state and especially cross-tier transitions remain limited. Low-efficiency states rarely achieve substantial upward shifts, and adjustments in high-efficiency states mainly occur through gradual movements to adjacent tiers. These patterns suggest that efficiency improvement is not the result of short-term policy shocks but is deeply shaped by existing resource allocation structures, organizational arrangements, and institutional environments (44). The inherent rigidity of the healthcare system in areas such as personnel training, infrastructure development, and service network organization generates strong path persistence (45). The relative stability of efficiency rankings therefore reflects the long-term embeddedness of institutional and structural factors.

The stationary distribution results indicate that efficiency at the national level does not converge toward a single equilibrium state. Instead, a long-term stratified structure persists across low, medium, and high efficiency tiers. Each state occupies a stable proportion under long-run limiting conditions. The low-efficiency state exhibits strong persistence, while the high-efficiency state, though representing a relatively smaller share, does not show signs of being absorbed through convergence. It should be emphasized that the “club” structure identified in this study is not defined in the strict sense of parametric convergence theory. Rather, it refers to a stratified configuration endogenously formed through high state persistence and limited cross-tier mobility within a dynamic transition framework (46). The coexistence of multiple tiers in the stationary distribution implies that, under the existing institutional and regional development framework, high-efficiency regions do not automatically induce systemic improvements in low-efficiency regions (38). Efficiency disparities therefore display structural persistence.

The stage-specific analysis further reveals temporal heterogeneity in efficiency evolution. Although strong state persistence is observed within each period, the stationary distribution structures differ across stages (47). It is important to note that the stage-specific stationary distributions reflect the relative efficiency ranking within each period under limiting conditions rather than changes in absolute efficiency levels (48). Accordingly, the higher share of low-efficiency states in the later stage does not necessarily imply overall performance deterioration (49). Instead, it may reflect the gradual consolidation of efficiency tiers in a context characterized by moderated resource expansion and a more stable institutional framework. These findings suggest that efficiency evolution is not a monotonic process of continuous improvement but is shaped by multiple factors, including macroeconomic conditions, the pace of healthcare reform, and structural adjustments in resource allocation (50).

The spatial Markov analysis further uncovers spatial conditional dependence in efficiency dynamics. After controlling for the initial efficiency state, the neighborhood efficiency environment continues to exert a significant influence on provincial transition probabilities. Provinces located within high-efficiency neighborhoods exhibit a higher likelihood of upward mobility from the low-efficiency state compared with similar provinces situated in low-efficiency neighborhoods. At the same time, high-efficiency states demonstrate stronger persistence within clusters of high-performing regions. These findings indicate that efficiency evolution is not an isolated process but is embedded within regional interaction structures (51). Similarities in institutional practices, resource allocation patterns, and governance experiences among neighboring provinces may reinforce clustering and persistence of efficiency states through mechanisms such as spatial spillovers and policy diffusion (52). As a result, efficiency club structures are strengthened not only over time but also across space.

The transition equation results indicate asymmetric associations between resource allocation structures, economic development, and upward versus downward efficiency mobility. The configuration of hospital beds relative to personnel is significantly associated with upward transitions (53), whereas personnel scale indicators are more prominently related to downward movements (36). It should be noted that the transition equations are not intended to establish strict causal relationships. Rather, they identify systematic correlations between changes in efficiency states and underlying resource structure conditions (54). These findings provide empirical insights into the factor constraints underlying efficiency mobility and lay the groundwork for future research employing stronger identification strategies.

Overall, the long-term evolution of provincial healthcare resource allocation efficiency in China is characterized by the coexistence of path dependence, structural stratification, and spatial conditional dependence. Efficiency improvement does not follow a pattern of natural convergence but instead reflects gradual adjustment within the constraints of existing institutional and regional structures (55). This implies that, even in the context of sustained expansion in resource inputs, disparities in marginal returns across regions may persist if the underlying efficiency structure is not concurrently optimized (56). The stability of efficiency stratification points to structural constraints and institutional inertia within the public health resource allocation system (57). These long-term dynamics carry significant implications for achieving balanced regional healthcare development and advancing health equity objectives.

## 6 Conclusion

Based on provincial panel data from 2001 to 2020, this study systematically examines the structural characteristics of healthcare resource allocation efficiency in China from the perspectives of dynamic evolution and spatial interdependence. The findings indicate pronounced path dependence in provincial efficiency, with state transitions occurring primarily through incremental movements between adjacent tiers and a low probability of cross-tier mobility. Over the long term, the evolution of efficiency results in the coexistence of low-, medium-, and high-efficiency tiers, forming a stratified structure. This stationary configuration suggests that efficiency disparities do not converge naturally over time but instead reflect gradual adjustments constrained by existing institutional arrangements, resource allocation structures, and regional development patterns. Short-term reforms or episodic policy interventions in individual regions are therefore unlikely to fundamentally alter the long-term structure of interprovincial efficiency rankings. These findings suggest that future healthcare planning should not rely solely on within-province adjustment. Given the spatial interdependence in provincial efficiency performance, greater attention should be paid to cross-regional coordination, institutional learning, and the alignment of healthcare resource allocation across neighboring regions.

The stage-specific analysis reveals that stationary distributions vary across development phases, reflecting the influence of macroeconomic conditions, fiscal capacity, and the pace of healthcare reform on the trajectory of efficiency evolution. The spatial Markov results further demonstrate significant spatial conditional dependence in efficiency transitions. High-efficiency neighborhood environments increase the likelihood of upward mobility from low-efficiency states, whereas clusters of low-efficiency regions reinforce state persistence. This spatial interaction mechanism suggests that efficiency stratification is not only characterized by temporal path dependence but is also embedded within regional structures, leading to the continuation of stratification through interregional interactions.

From a policy perspective, efficiency improvement cannot be achieved solely through expanding resource inputs or optimizing governance within individual regions. Regional coordination mechanisms, cross-regional alignment of resource allocation, and the diffusion of institutional experience may play a critical role in mitigating stratification and promoting dynamic mobility. In particular, the spatial reinforcement mechanism identified in this study points to the importance of stronger cross-provincial coordination, regional resource-sharing arrangements, and institutional learning across provinces. Optimizing the configuration of healthcare service networks and strengthening interregional institutional learning can enhance the upward mobility of low-efficiency regions and reduce the risk of structural entrenchment.

Overall, this study advances the understanding of healthcare resource allocation efficiency in China by integrating dynamic and spatial perspectives and situating efficiency analysis within a long-term structural evolution framework. The findings provide empirical support for identifying the structural roots of regional disparities in healthcare development. Future research may further explore the deeper mechanisms underlying efficiency stratification and evolution by employing more refined spatial weighting schemes and stronger causal identification strategies.

This study also has several limitations. First, the analysis is conducted at the provincial level and therefore cannot capture variation within provinces. Second, the DEA-based efficiency scores depend on the selected input–output indicators and reflect relative rather than absolute efficiency. Third, the Markov and spatial Markov framework is mainly used to describe dynamic patterns and spatial conditional dependence, rather than to identify causal relationships. Future research could build on this analysis by using more disaggregated data, alternative spatial weighting schemes, and stronger causal identification strategies.

## Data Availability

The original contributions presented in the study are included in the article/Supplementary material, further inquiries can be directed to the corresponding author.
